# Supplementary figures and images for: Ninjin’yoeito reduces fatigue-like conditions by alleviating inflammation of the brain and skeletal muscles in aging mice
Source: PLoS One. 2024 May 20;19(5):e0303833. doi: 10.1371/journal.pone.0303833 (PMC11104581; doi:10.1371/journal.pone.0303833)

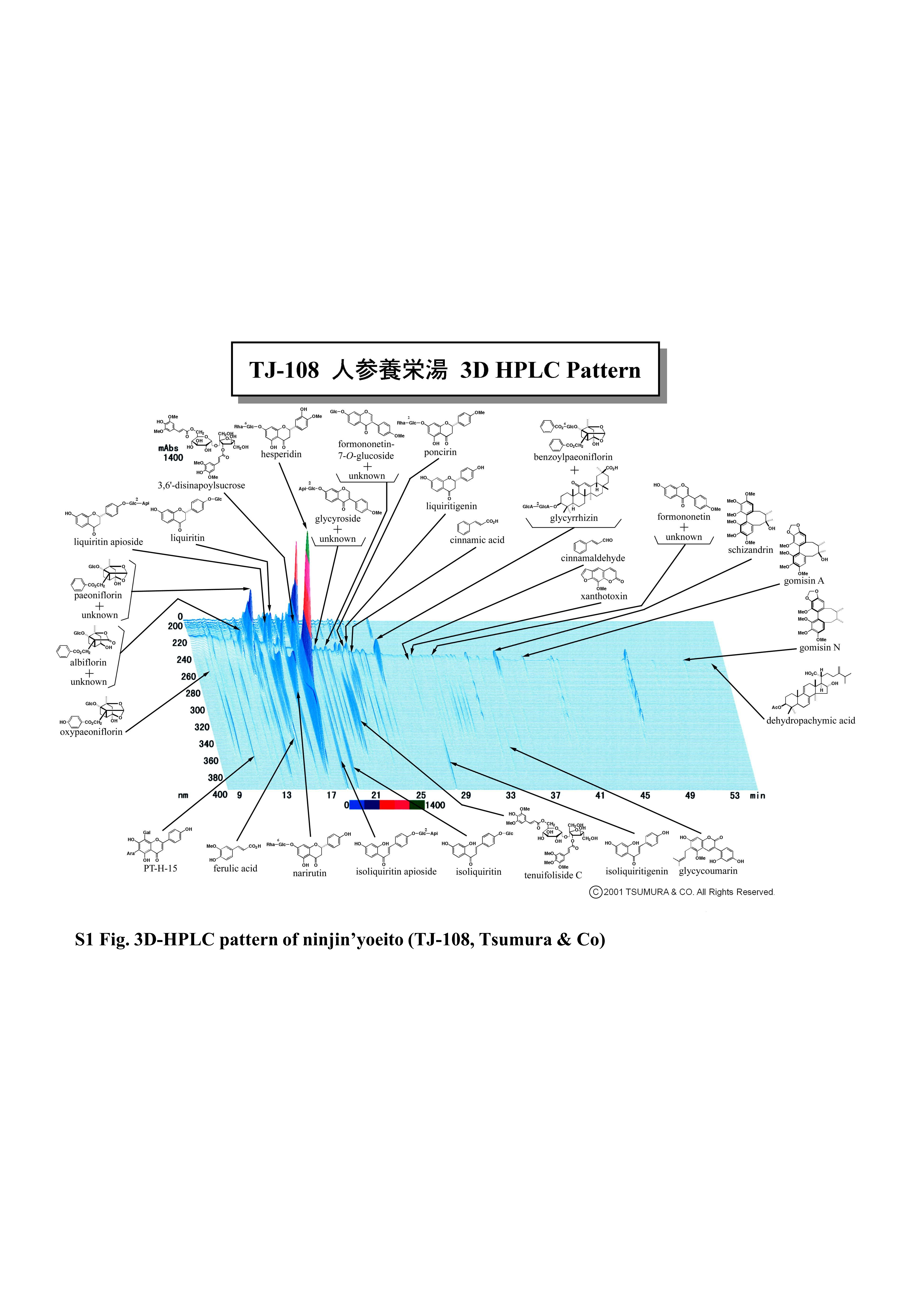

Supplement: S1 Fig — (TIFF) [file pone.0303833.s003.tiff]

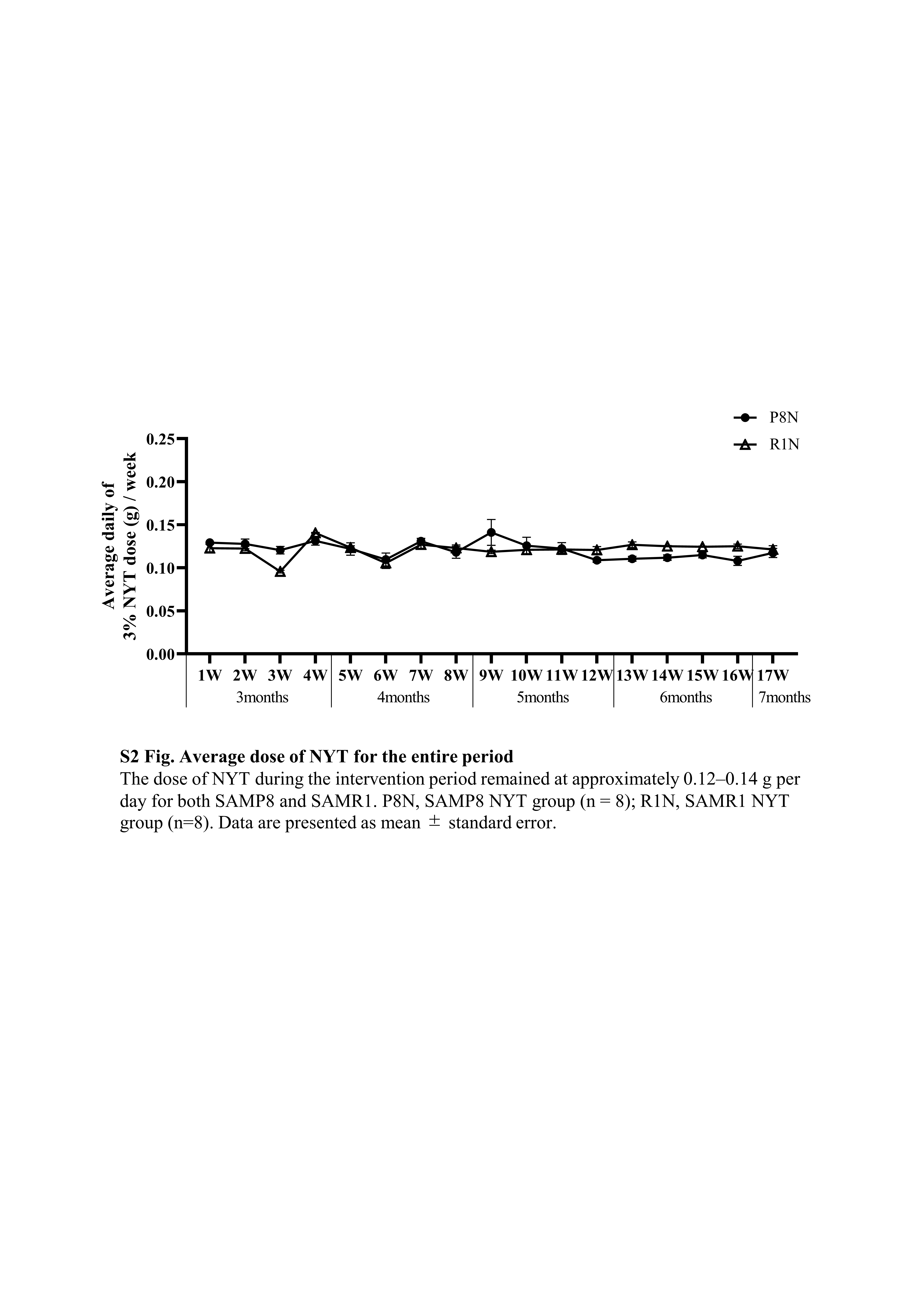

Supplement: S2 Fig — (TIFF) [file pone.0303833.s004.tiff]

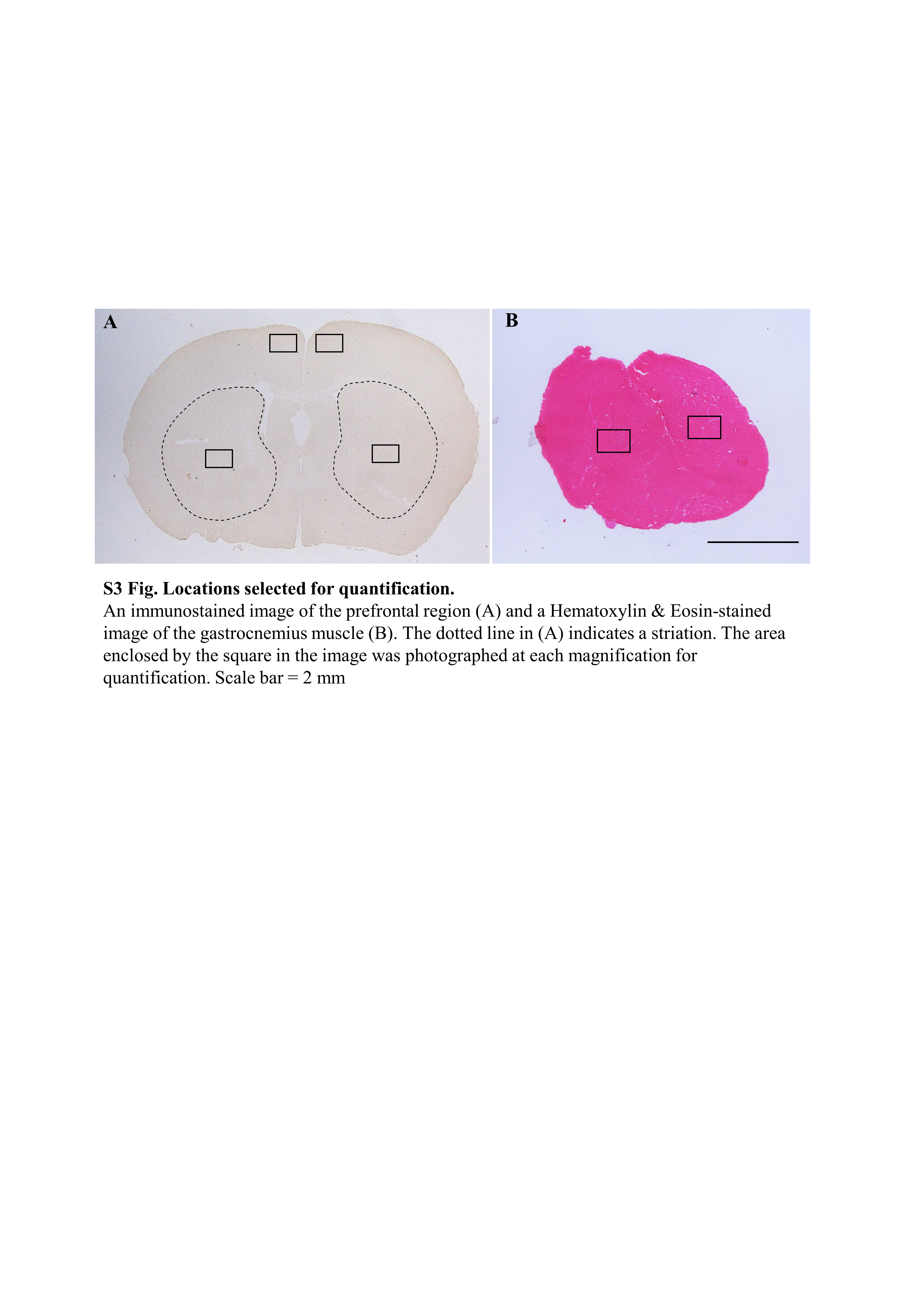

Supplement: S3 Fig — (TIFF) [file pone.0303833.s005.tiff]

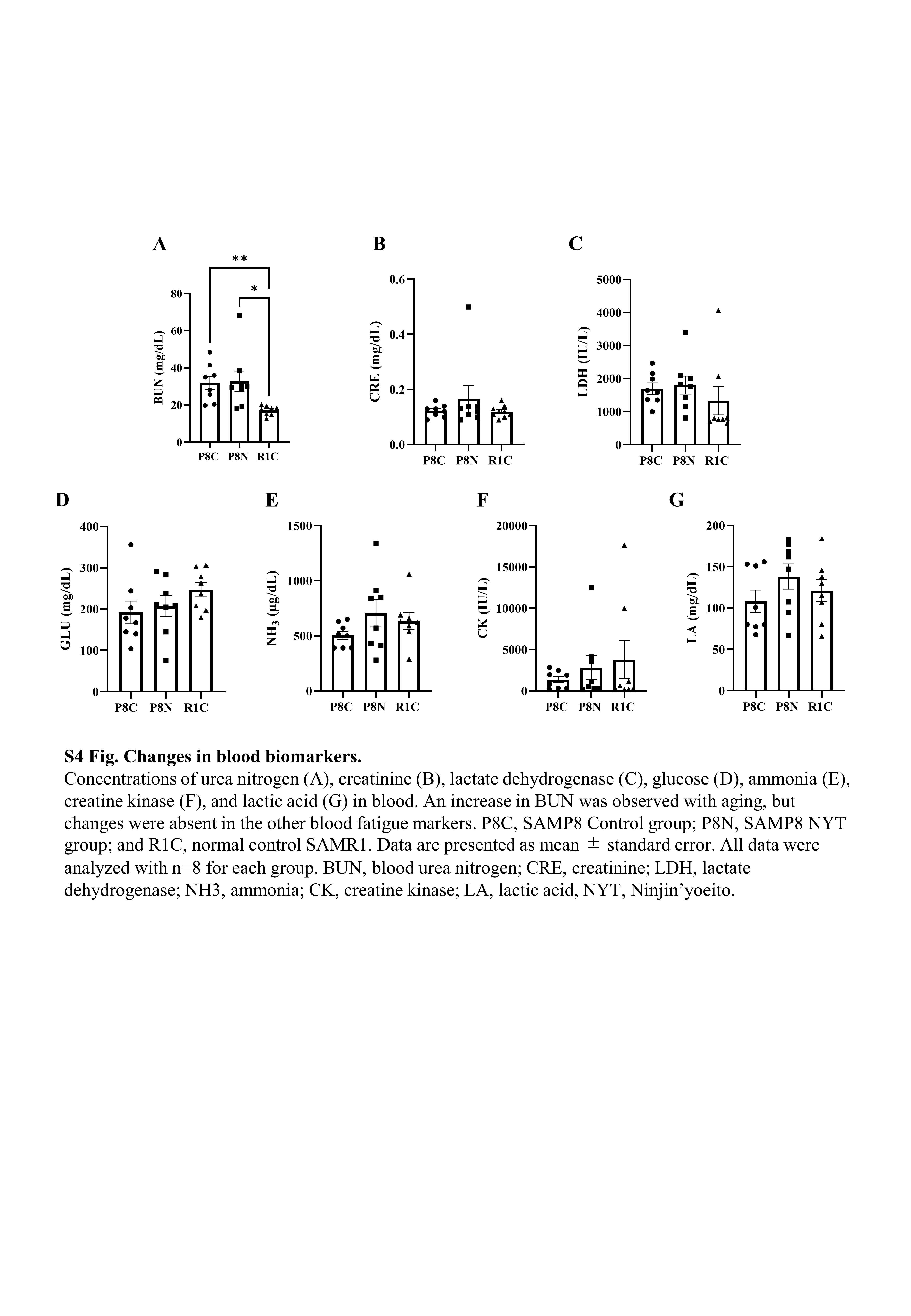

Supplement: S4 Fig — (TIFF) [file pone.0303833.s006.tiff]
