# Supplementary material for: Ninjin’yoeito reduces fatigue-like conditions by alleviating inflammation of the brain and skeletal muscles in aging mice
Source: PLoS One. 2024 May 20;19(5):e0303833. doi: 10.1371/journal.pone.0303833 (PMC11104581; doi:10.1371/journal.pone.0303833)
Supplement: S1 File — (DOCX) [file pone.0303833.s007.docx]

**Original underlying images for all Western blot.**

**IL-1β for brain (Fig.5 C)**


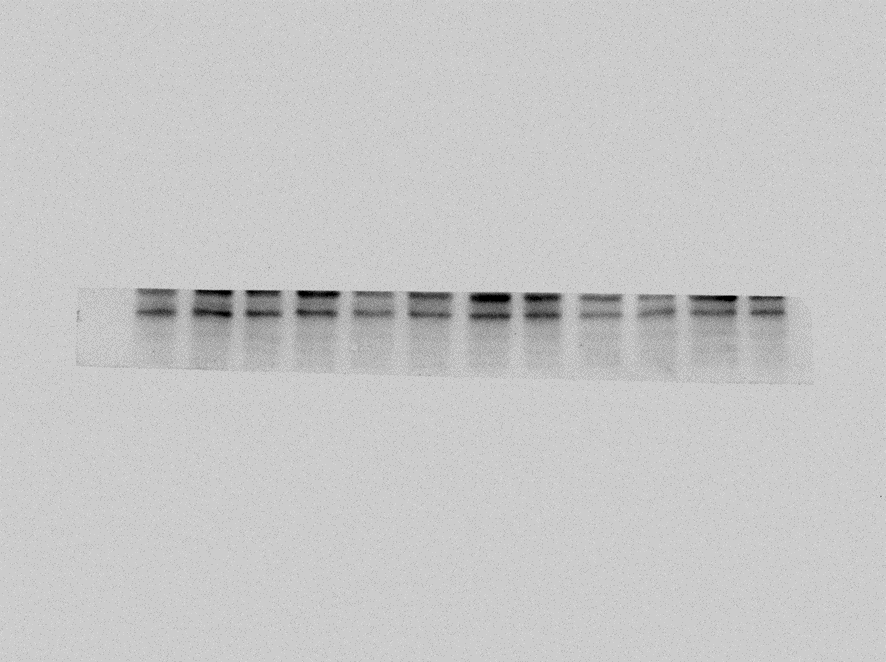


**―25 kDa (IL-1β)**

**35 kDa－**

**25 kDa－**

**20 kDa－**

**５**

**６**

**７**

**８**

**９**

**10**

**11**

**12**

**1**

**２**

**３**

**４**

**IL-1RA for brain (Fig.5 C)**


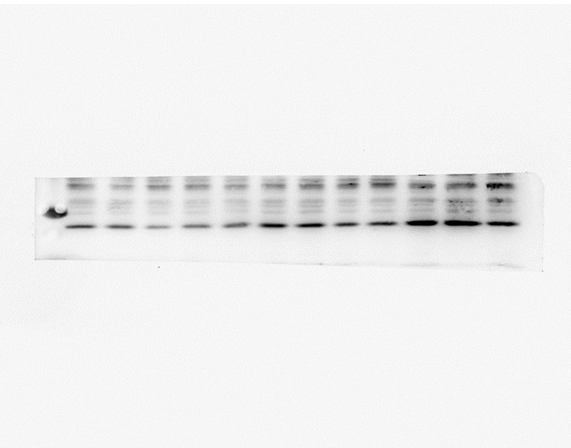


**―20 kDa (IL-1RA)**

**35 kDa－**

**20 kDa－**

**25 kDa－**

**９**

**10**

**11**

**12**

**５**

**６**

**７**

**８**

**1**

**２**

**３**

**４**

**Iba-1 for brain (Fig.5 C)**


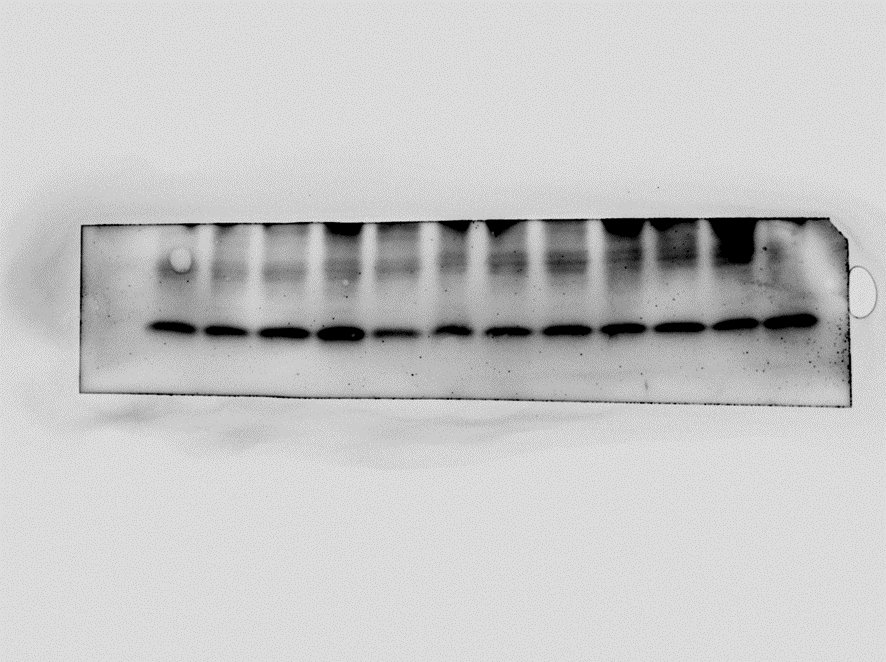


**35 kDa－**

**17 kDa－**

**25 kDa－**

**―16 kDa (Iba-1)**

**９**

**10**

**11**

**12**

**５**

**６**

**７**

**８**

**1**

**２**

**３**

**４**

**11 kDa－**


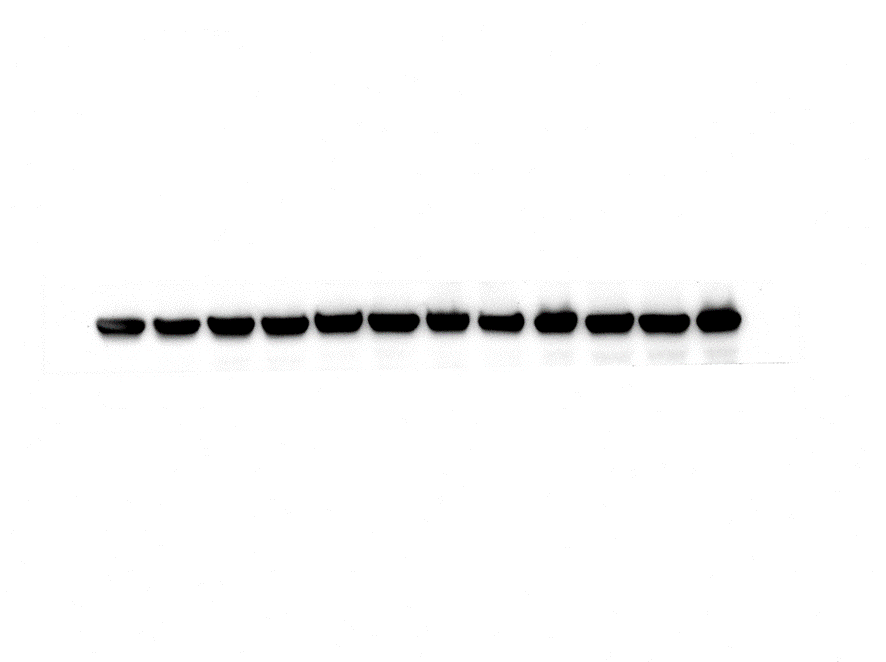
 **α-tubulin (Fig.5 a)**

**75 kDa－**

**―52 kDa (α-tubulin)**

**９**

**10**

**11**

**12**

**５**

**６**

**７**

**８**

**1**

**２**

**３**

**４**

**Western blot**

**1. SAMP 8 Cont No.1　2. SAMP 8 Cont No. 2　3. SAMP 8 Cont No.3　4. SAMP 8 Cont No.4**

**5. SAMP 8 NYT No.1　6. SAMP 8 NYT No.2　7. SAMP 8 NYT No.3　8. SAMP 8 NYT No.4**

**9. SAMR１No.1 10. SAMR１No.2 11. SAMR１No.3 12. SAMR１No.4**

**IL-1β for muscle (Fig. 6 C)**

**９**

**10**

**11**

**12**

**５**

**６**

**７**

**８**

**1**

**２**

**３**

**４**


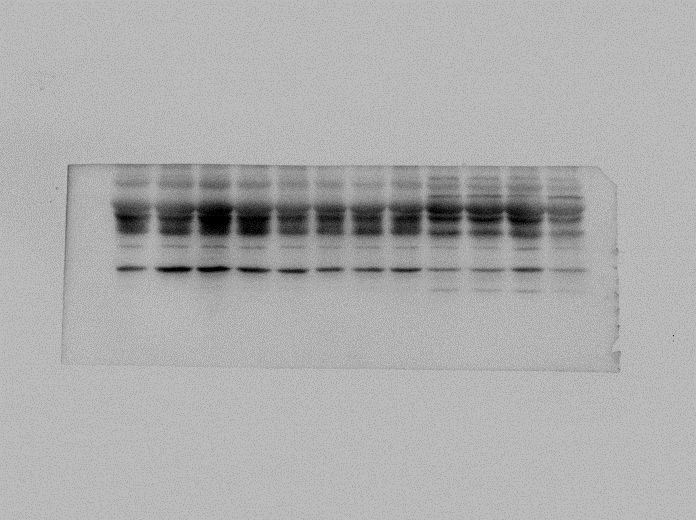


**―25 kDa (IL-1β)**

**35 kDa－**

**75 kDa－**

**25 kDa－**

**20 kDa－**

**IL-1RA for muscle (Fig.6 C)**


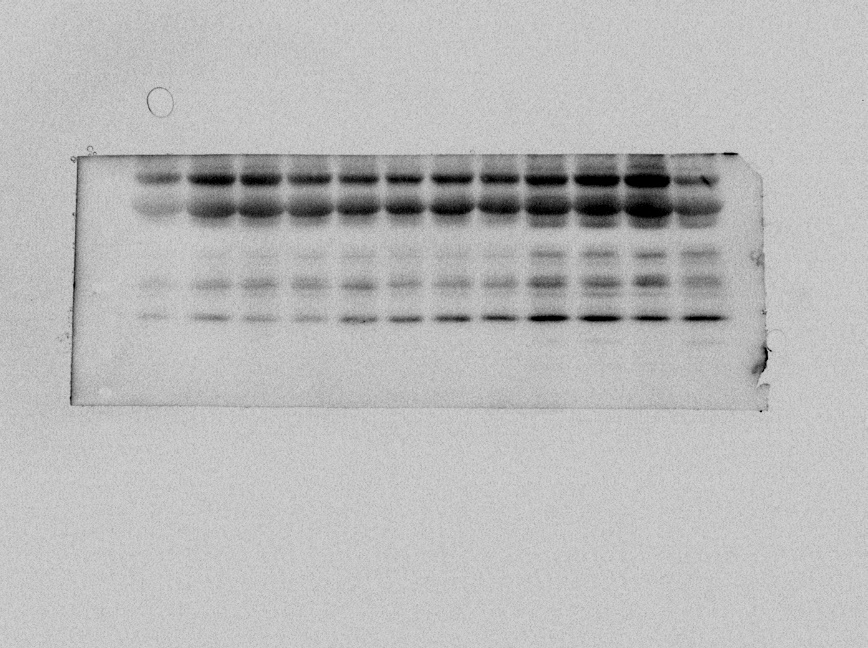


**75 kDa－**

**35 kDa－**

**20 kDa－**

**25 kDa－**

**―20 kDa (IL-1RA)**

**９**

**10**

**11**

**12**

**５**

**６**

**７**

**８**

**1**

**２**

**３**

**４**

**GAPDH for muscle** **(Fig.6 C)**


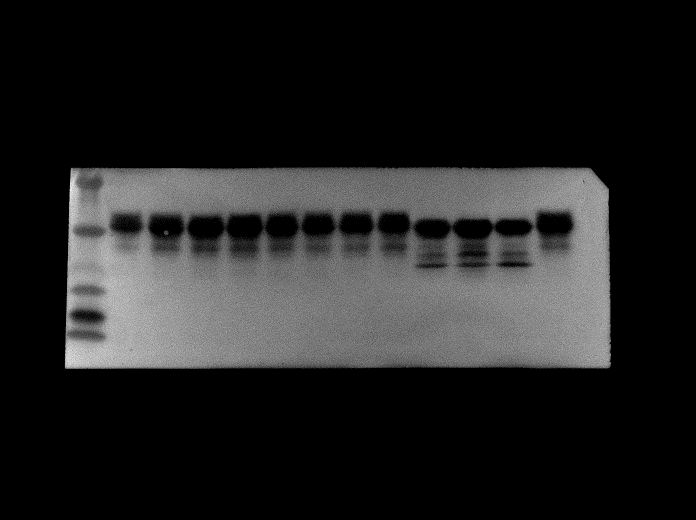


**35 kDa－**

**―38 kDa (GAPDH)**

**25 kDa－**

**20 kDa－**

**17 kDa－**

**Western blot**

**1. SAMP 8 Cont No.1　2. SAMP 8 Cont No. 2　3. SAMP 8 Cont No.3　4. SAMP 8 Cont No.4**

**5. SAMP 8 NYT No.1　6. SAMP 8 NYT No.2　7. SAMP 8 NYT No.3　8. SAMP 8 NYT No.4**

**9. SAMR１No.1 10. SAMR１No.2 11. SAMR１No.3 12. SAMR１No.4**
