# Supplementary material for: Ninjin’yoeito reduces fatigue-like conditions by alleviating inflammation of the brain and skeletal muscles in aging mice
Source: PLoS One. 2024 May 20;19(5):e0303833. doi: 10.1371/journal.pone.0303833 (PMC11104581; doi:10.1371/journal.pone.0303833)
Supplement: Attachment — Submitted filename: renamed_441eb.pdf [file pone.0303833.s009.pdf]

This paper is very interesting as it shows that ninjin'yoeito improves age-related inflammatory conditions in both the central and peripheral tissues and reduces fatigue. It has also been shown that SAMP8 can serve as a model animal for fatigue, making this an important paper that will contribute to the development of future research. In addition, as this study showed that a treatment method using ninjin'yoeito can improve age-related fatigue, it is expected that it will be applied to recovery from fatigue in the elderly.

I would like to ask some questions because there are some things that are unclear.

Materials and methods

Animals:

1. There is a statement that SAMP8 mice were purchased at 3 months old, but wouldn't the breeding environment have changed significantly?
2. Mice are kept in groups of 2-3 per cage, but wouldn't the meaning of the breeding environment be different if two mice are housed one-on-one versus three or more mice to form a society?

NTY preparation:

3. The authors divided the experiment groups into four. This is often a factor that confounds the interpretation of results. There are difficulties in using younger SAMR for comparison. The authors use it differently depending on the purpose, but except for Figure 4 the control is R1C. It is thought that R1N should also be included in the graph data.
4. There is a statement that it is mixed into the feed at a concentration of 3% (w/w), but is this the appropriate concentration? Are the density settings quoted from somewhere? Please indicate the basis for setting the concentration.
5. Is it correct to understand that SAMR is a mouse of normal age? Compared to SAMP8, when mice of the same age are used in experiments, SAMR, which is a younger mouse, seems to have a better appetite. If this is the case, it is thought that the dose of NTY may be different from that of SAMP8 since it is administered ad libitum. Does that mean that the problem was corrected by correcting the weight?

Quantiative analysis of immunostained sections, and S3 Figure B:

6. Does the region selected for analysis represent the center of the striatum? From S3 Figure A, the staining is unclear and the site is unclear.

The striatum also has site-specific roles. For example, the ventral striatum (VS) is involved in motivated behavior. If you chose the central part of the striatum, what was the reason? Also, why did they choose the striatum in the brain? Other issues related to exercise include neural circuits with the basal ganglia, motor control, and reduction in the amount of activity. Please discuss why

you chose the striatum, along with the results of behavioral analysis.

7. Immunohistochemical staining was used to distinguish between fast and slow muscles. Was there any deviation in the distribution? For example, there are many slow-twitch muscles in the deep layers (close to bones), and on the contrary, there are many fast-twitch muscles in the superficial layers. Why did you choose the part you examined?

Results

p.19, L.325-327:

8. Is the peak weight of SAMR1 at 6 months of age, coinciding with the peak of SAMP8? Since SAMP8 accelerates aging, it may have reached its peak earlier. Is it appropriate to compare SAMP and SAMR at this month of age?

p.21, L.356-360:

9. Why did only corticosterone show high levels? What is the difference from other blood fatigue markers? It is stated in the discussion that it reflects the period immediately after exercise, but how long does corticosterone reflect fatigue? Or are these differences based on differences in the principles of fatigue? Please explain the difference.

p.23, L.397- p.24, L.406:

10. The results of administering NYT to SAMR and its control group are described here. If this result was shown first, it would be easier to understand that there is no RIN in the graph of Figure 1-3. I would like to suggest that the order of the structure of the paper be changed.

p.24, L405-406:

11. SAMR1 has no effect of NYT, while SAMP8 has been shown to have the effect. Is there a difference in fatigue between young and old people? Also, are there differences in the mechanism of fatigue recovery? Please answer about them.

Figure 3:

12. Although the areas of muscle fibers are compared, the difference in number is more obvious than the area, especially in the photo of “D” Type 1 fibers. Isn't it a difference in area due to a difference in number?

Also, am I correct in my understanding that P8N can maintain the area of fast-twitch muscles? Analysis of the expression of switch proteins such as PGC1α can reveal the slow muscle fibers associated with aging.

Figure 4:

13. Regarding “K”, fast twitch fibers generally replace slow twitch fibers as people age in the human. In mice treated with NYT, this did not occur and the number of fast-twitch muscles was maintained. Does it have an anti-aging effect on muscles?

Minor comments:

p.12, L.188：

I referred to literature [18], but there is no mention of the brightness of the room. Please indicate the measurement conditions.

P.19, L.317:

$P<0.05$: Shouldn't "P" be lowercase? $p<0.05$

p.25, L.427, 429:

Fig 5D, F, $p<0.01$, Fig 5D, F, $p<0.05$ Isn't it "E" instead of "F"? please confirm.

Materials and methods, Behavioral test:

Mice are nocturnal, so isn't it more reliable to perform behavioral tests at night?

Materials and methods, Histology and immunohistochemistry:

There is no description of the location of the coronal section of the brain. It is usually written as how many mm forward from bregma.

Figure 1:

The text compares SAMR and SAMP. However, the way graph "B" is drawn is not drawn as graph "D." Please check the graphs of other figures again. It is a horizontal bar that shows the p value.

Figure 4:

Regarding “A”, there was no difference between the three groups, is there any particular need to show it as a photograph? Also, if you want to show it, why not show the positive cells with an arrow head?
