# Supplementary material for: Ninjin’yoeito reduces fatigue-like conditions by alleviating inflammation of the brain and skeletal muscles in aging mice
Source: PLoS One. 2024 May 20;19(5):e0303833. doi: 10.1371/journal.pone.0303833 (PMC11104581; doi:10.1371/journal.pone.0303833)
Supplement: Attachment — Submitted filename: Response_to_Reviewers.docx [file pone.0303833.s010.docx]

Response to Reviewer #1

1．SAMP8 is aggressive, 2 or 3 mice in one cage, if is there the mice fight even hurt? If the mice fight each other, it would increase the fatigue, and if hurt, it might increase the inflammation status. And, the author also found the intake NYT or foods for each mice were different.

**Answer:**

We thank the reviewer for this valuable comment. SAMP8 mice were regularly monitored throughout the experiment to prevent injury, and none of the mice were harmed. The results of the behavioral and histological analyses did not show any significant differences between the groups, indicating no effect. The number of animals housed did not appear to have an effect, as both groups were housed under the same conditions, with two or three animals per cage in both the NYT-treated and non-treated groups. As the current study focused on whether NYT reduces age-related fatigue, further research is needed to determine the effective dosage of NYT for clinical application.

2．the survival mice on the 7-month age should be explained

**Answer:**

We thank the reviewer for this valuable comment. In this study, no individuals died in SAMP8 at 7 months of age. This finding is consistent with previous studies that have reported that SAMP8 mice start to die at the age of 8 months (PMID: 33388314, PMID: 37710278, PMID: 37182575).

***Materials and Methods, NYT preparation***

**Original, P11, line 167-168**

・・・within 2h. These endpoints were not reached during the experimental period.

**Revised, P11, line 167-169**

・・・within 2 h. These endpoints were not reached during the experimental period. Additionally, none of the individuals died from either SAMR1 or SAMP8 before the age of 7 months.

3. the dosage of NYT of 0.3&times;food, how to get this dosage

**Answer:**

We thank the reviewer for this valuable comment. In this experiment, the mice were fed a diet consisting of 3% NYT mixed with regular food. Food intake was measured weekly to calculate the NYT dose, which was determined by multiplying food intake by 0.03.

Response to Reviewer #2

Materials and Methods

Animal:

1．There is a statement that SAMP8 mice were purchased at 3 months old, but wouldn’t the breeding environment have changed significantly?

**Answer:**

We thank the reviewer for this valuable comment. Mice were purchased at 7 weeks of age and reared until they were 3 months old. The rearing environment remained consistent throughout the study, and it is unlikely that any environmental changes had a significant impact on the results. The manuscript contained an error regarding the age and rearing of 7-week-old SAMP8 and SAMR1 mice. The revised text is as follows:

***Materials and Methods***

**Original, P8, line 119**

Sixteen 3-month-old male SAMP8 (weight: 26.5 ± 2.0 g) and ・・・・・・

**Revised, P8, line 119**

Sixteen 7-week-old male SAMP8 (weight: 26.5 ± 2.0 g) and ・・・・・・

2．Mice are kept in groups of 2-3 per cage, but wouldn’t the meaning of the breeding environment be different if two mice are housed one-on-one versus three or more mice to form a society?

**Answer:**

We thank the reviewer for this valuable comment. At Kagoshima University, the rearing standard is to maintain two animals instead of three. There were no significant differences in locomotion, body weight, food intake, or activity between individuals maintained with three animals and those maintained with two animals. Additionally, each group included individuals reared with two animals. The present study focused on age-related fatigue, and it is unlikely that the number of animals reared would have an effect.

NTY preparation:

3．The authors divided the experiment groups into four. This is often a factor that confounds the interpretation of results. There are difficulties in using younger SAMR for comparison. The authors use it differently depending on the purpose, but except for Figure 4 the control is R1C. It is thought that R1N should also be included in the graph data.

**Answer:**

We thank the reviewer for this valuable comment. SAMP8 mice are frequently used in aging studies, with SAMR1 serving as a control group. This study investigated whether SAMP8 is an appropriate model for the development of fatigue-like symptoms associated with aging. Therefore, it was necessary to compare 7-month-old SAMP8 with SAMR1 of the same age, and SAMR1 was used in this study. Additionally, a group of mice treated with NYT was used to verify whether NYT affected normal mice. The study found no significant differences in the evaluations between groups R1C and R1N. Subsequently, we compared three groups: the SAMP8 intervention group 'P8N,’ the non-intervention group 'P8C,’ and 'R1C' as the normal control group. The structure of the paper has been reorganized for better comprehension by the reader.

***Results***

**Original, P19‒20, lines 322‒348**

NYT improves aging-related symptoms

The effects of NYT on aging-related weight loss, anorexia, and loss of locomotor function were examined. Body weight at 6 and 7 months of age did not differ among the three groups (Fig 1A); the rate of weight loss at 7 months of age was significantly higher in the two SAMP8 groups than in the SAMR1 group which exhibited a peak weight at 6 months of age (p < 0.01). However, the rate of weight loss was significantly lower in the SAMP8 NYT group than in the SAMP8 Control group (Fig 1B, p < 0.05). Furthermore, the SAMP8 NYT group had the highest average daily food intake (Fig 1C). In the Rotarod test, which evaluates motor function, the SAMP8 Control group had a significantly shorter walking time than did the SAMR1 group (Fig 1D, p < 0.01). Similarly, the SAMP8 Control group was significantly weaker than the SAMR1 group in grip strength (Fig 1E, p < 0.01). However, the SAMP8 NYT group showed significant improvement over the SAMP8 Control group in walking time and grip strength in the Rotarod test (Fig 1D, E, p < 0.01). These results suggest that NYT administration improves weight loss, anorexia, and motor function associated with aging.

Fig 1. Effect of NYT on age-related weight loss, anorexia, and motor function decline.

・・・・.

**Revised, P19‒20, lines 328-348**

To determine whether NYT alters the physical function of SAMR1 mice with normal aging, SAMR1 mice treated with and without NYT were compared. Body weight, walking time in the Rotarod test, grip strength, treadmill fatigue test, gastrocnemius weight, and cross-sectional area did not show significant changes after NYT treatment (Fig 1A, C, D, E, H, I, J). Ninjin’yoeito administration increased food intake and blood corticosterone levels and decreased resting time in the open field test; however, the differences were not statistically significant (Fig 1B, F, G). These results suggest that NYT does not enhance the physical function of SAMR1.

Fig 1. Effects of NYT on body weight, appetite, physical function, fatigue, and gastrocnemius muscle in SAMR1.

Body weight at 7 months of age (A), average daily food intake (B), Rotarod test results (C), grip strength results (D), treadmill fatigue test results (E), percentage increase in resting time in the open field test (F), blood corticosterone concentration (G), and gastrocnemius weight-to-weight ratio (H). Feeding NYT to SAMR1 mice had no significant effects on body weight, food intake, physical function, fatigue, or the gastrocnemius muscle. “R1C,” SAMR1 mice fed the Control diet (n = 8); “R1N,” SAMR1 mice fed NYT (n = 8). Data are shown as mean ± standard error. Scale bar = 150 μm.

***Discussion***

**Original, P33, line 563-**

・・・is effective for clinical applications. Second, we used only SAMP8 mice in this study. ・・・

**Revised, P33, line 569-573**

・・・is effective for clinical applications. Second, this study investigated the effects of NYT on SAMR1 in normal mice. These results confirmed that NYT administration did not alter SAMR1. Therefore, a comparison was made among the three groups: SAMR1 fed a normal diet was used as the normal control group, and SAMP8 was used as the group that was administered NYT or not. Third, we used only SAMP8 in this study.・・・

4．There is a statement that it is mixed into the feed at a concentration of 3% (w/w), but is this the appropriate concentration? Are the density settings quoted from somewhere? Please indicate the basis for setting the concentration.

**Answer:**

We thank the reviewer for this valuable comment.

1. Ninjin’yoeito ameliorated PPE-induced pulmonary emphysema and anxiety/depressive-like behavior in aged C57BL/6J mice
2. Ninjin'yoeito Ameliorates Skeletal Muscle Complications in COPD Model Mice by Upregulating Peroxisome Proliferator-Activated Receptor γ Coactivator-1α Expression

The concentrations were determined with reference to these two references.

5．Is it correct to understand that SAMR is a mouse of normal age? Compared to SAMP8, when mice of the same age are used in experiments, SAMR, which is a younger mouse, seems to have a better appetite. If this is the case, it is thought that the dose of NTY may be different from that of SAMP8 since it is administered ad libitum. Does that mean that the problem was corrected by correcting the weight?

**Answer:**

We thank the reviewer for this valuable comment. The experiment was conducted using SAMP8 and SAMR1 mice of the same age. When the results were rechecked, the food intake of SAMP8 was incorrect, resulting in different graphs. We apologize. The daily food intake in this experiment was P8N: 4.021 ± 0.067 g and R1N: 4.049 ± 0.075 g (mean ± standard error). As NYT has an appetite-stimulating effect, it is possible that the SAMP8s treated with NYT had a higher food intake and that the NYT-treated SAMP8 and SAMR1 mice had similar food intake; the dose of NYT did not differ between SAMP8 and SAMR1 mice. We replace the following graph.

**Original**　　　　　　**Revised**

Quantiative analysis of immunostained sections, and S3 Figure B:

3．Does the region selected for analysis represent the center of the striatum? From S3 Figure A, the staining is unclear and the site is unclear.

The striatum also has site-specific roles. For example, the ventral striatum (VS) is involved in motivated behavior. If you chose the central part of the striatum, what was the reason? Also, why did they choose the striatum in the brain? Other issues related to exercise include neural circuits with the basal ganglia, motor control, and reduction in the amount of activity. Please discuss why you chose the striatum, along with the results of behavioral analysis.

**Answer:**

We thank the reviewer for this valuable comment. In this study, we assessed the association between fatigue and brain inflammation. The study also focused on microglia, which are involved in inflammation in the brain; western blot analysis was performed using the prefrontal cortex, which includes the striatum in addition to the cortex, and the results showed that microglial protein levels did not change after NYT administration. In addition to the western blot analysis, IHC analysis was performed on similar areas, including the striatum. The results showed no changes in microglia, as observed using western blotting.

Therefore, we believe that the striatal function is important. In the present study, we did not focus on the specific function of the striatum but rather assessed microglia in the prefrontal cortex. Therefore, the central part of the striatum was selected as a quantitative site. Motivation was not assessed in the present study; however, there was no significant difference in the rate of decrease in total activity at seven months of age. Future studies should focus on striatal regions and motivation in addition to these limitations. We have replaced the images with quantitative brain locations in the Supplementary Figure. Dotted lines in the image indicate the striatum.

**Original, P34, line 588-**

・・・after NYT administration in the brain. Despite these limitations,

**Revised, p35, line 602-608**

・・・after NYT administration in the brain. This study investigated inflammation in the brain using the prefrontal cortex, including both the cortex and striatum, using western blotting. Immunohistochemical analysis was also conducted on the cortex and striatum of the prefrontal cortex. The study did not focus on the specific functions of the striatum but rather assessed microglia in the prefrontal cortex. Future research should aim to investigate striatal regions and motivation. Despite these limitations,

**Supplementary Figure**

**Original**　　　　　　　　　　　　　　　　　**Revised**

**A**

**A**

7. Immunohistochemical staining was used to distinguish between fast and slow muscles. Was there any deviation in the distribution? For example, there are many slow-twitch muscles in the deep layers (close to bones), and on the contrary, there are many fast-twitch muscles in the superficial layers. Why did you choose the part you examined?

**Answer:**

We thank the reviewer for this valuable comment. The gastrocnemius muscle has Type 1 and Type 2 fibers in the deep layers and only Type 2 fibers in the superficial layers. Skeletal muscle atrophy due to aging is known to occur in Type 2 fibers significantly. The deep layer was selected in the present study to investigate whether NYT affects the myofiber type per fiber type in aging skeletal muscles.

Results

p.19, L.325-327:

8. Is the peak weight of SAMR1 at 6 months of age, coinciding with the peak of SAMP8? Since SAMP8 accelerates aging, it may have reached its peak earlier. Is it appropriate to compare SAMP and SAMR at this month of age?

**Answer:**

We thank the reviewer for this valuable comment. In the present study, body weight was measured at defined points during the intervention period, in addition to condition observations. The results indicated that SAMP8 mice exhibited a peak body weight at six months of age and subsequently began to lose weight, which is consistent with our previous study (ref. 18). In contrast, SAMR1 body weight is likely to increase after 7 months. In the present study, 7-month-old SAMR1s were used in experiments to compare them as a normal aging group against 7-month-old age-accelerated mice.

p.21, L.356-360:

9. Why did only corticosterone show high levels? What is the difference from other blood fatigue markers? It is stated in the discussion that it reflects the period immediately after exercise, but how long does corticosterone reflect fatigue? Or are these differences based on differences in the principles of fatigue? Please explain the difference.

**Answer:**

We thank the reviewer for this valuable comment. A possible reason for the high corticosterone levels could be that corticosterone increases with aging in addition to fatigue. Higher corticosterone levels have been reported in aging mice than in young mice (PMID: 32504071). Several previous studies measured other blood markers of fatigue after forced exercise. Therefore, the timing of blood sampling in the present study may not match the blood markers of age-related fatigue.

It is not clear whether and to what extent elevated corticosterone levels are indicative of fatigue. As the present study focused on aging-associated fatigue, it is possible that aging-associated corticosterone elevation may be associated with fatigue. Few animal studies have been conducted on age-derived fatigue. Therefore, the elevated corticosterone levels observed in this study may be an indicator of age-related fatigue.

p.23, L.397- p.24, L.406:

10. The results of administering NYT to SAMR and its control group are described here. If this result was shown first, it would be easier to understand that there is no RIN in the graph of Figure 1-3. I would like to suggest that the order of the structure of the paper be changed.

**Answer:**

We thank the reviewer for this valuable comment. NYT-treated and untreated SAMR1 cells were compared at the beginning of the experiment. We added the limitation that NYT had no effect on SAMR1 in normal mice; therefore, we compared the three groups. Further details are provided in the third section.

p.24, L405-406:

11. SAMR1 has no effect of NYT, while SAMP8 has been shown to have the effect. Is there a difference in fatigue between young and old people? Also, are there differences in the mechanism of fatigue recovery? Please answer about them.

**Answer:**

We thank the reviewer for this valuable comment. Younger people tend to feel fatigued after engaging in strenuous physical activity or using digital devices for long periods. Younger and older individuals experience fatigue differently. By contrast, older adults feel fatigued as they go about their daily lives. The mechanisms underlying fatigue are complex and include both physical and mental factors. Further research is required to understand these experiences fully. However, it is evident that inflammation contributes to fatigue and that older individuals with reduced resistance to inflammation may be more susceptible to chronic fatigue in their daily lives. Therefore, there may be variations in fatigue recovery between younger and older individuals.

Figure 3:

12. Although the areas of muscle fibers are compared, the difference in number is more obvious than the area, especially in the photo of “D” Type 1 fibers. Isn't it a difference in area due to a difference in number?

Also, am I correct in my understanding that P8N can maintain the area of fast-twitch muscles? Analysis of the expression of switch proteins such as PGC1α can reveal the slow muscle fibers associated with aging.

**Answer:**

We thank the reviewer for this valuable comment. The cross-sectional area of the gastrocnemius muscle was determined using fluorescence microscopy at 10× magnification and the cross-sectional areas of all Type 1 fibers in the image. The cross-sectional area results were not divided by the number of fibers; therefore, it is unlikely that the number of fibers affected the area results. The muscle weight and cross-sectional area were examined to determine the association between fatigue and muscle atrophy. The results showed that the administration of NYT inhibited the atrophy of Type 2 fibers but did not change Type 1 fibers. This study tested whether NYT is effective in reducing age-related fatigue. Therefore, future research is needed to determine the effects of NYT on aging muscles and fibers other than the gastrocnemius muscles. The following text has been added to the Limitations section:

***Discussion***

**Original, P34, line 583-**

・・・・other muscles as well. Finally, it appears・・・・

**Revised, P34, line 593-597**

・・・・other muscles as well. Additionally, the administration of NYT prevented a reduction in the cross-sectional area of Type 2 fibers but did not affect the cross-sectional area of Type 1 fibers. Therefore, further investigations are required to determine the effect of NYT on each muscle fiber type in aging muscles. Finally, it appears・・・・

Figure 4:

13. Regarding “K”, fast twitch fibers generally replace slow twitch fibers as people age in the human. In mice treated with NYT, this did not occur and the number of fast-twitch muscles was maintained. Does it have an anti-aging effect on muscles?

**Answer:**

We thank the reviewer for this valuable comment. NYT has the potential to induce an anti-aging effect in muscles by ameliorating oxidative stress and inflammation, which increase with age. A previous study (Ref: 18) reported that NYT administration to SAMP8 improved motor function and grip strength and inhibited muscle atrophy. Additionally, one study reported that NYT administration inhibited disuse muscle atrophy. However, we are still researching the points you raised and are planning to conduct a comprehensive study in the future.

・Disuse muscle atrophy-improving effect of ninjin'yoeito in a mouse model

*Minor comments:*

p.12, L.188：

I referred to literature [18], but there is no mention of the brightness of the room. Please indicate the measurement conditions.

**Answer:**

We thank the reviewer for this valuable comment. The brightness of the lighting was 150–300 lux, which is the standard for lighting at the Kagoshima University Animal Experimental Facility. The following additions have been made.

**Original, P12, line 197**

・・・Tests were conducted between 10:00 a.m. and 5:00 p.m.

**Revised, P12, line 200–201**

・・・tests were conducted between 10:00 a.m. and 5:00 p.m. The brightness of the room lighting during the tests ranged from 150 to 300 lux.

P.19, L.317:

P<0.05: Shouldn't "P" be lowercase? p<0.05

**Answer:**

We thank the reviewer for this valuable comment. We have corrected this as suggested.

**Original, P19, line 317**

・・・・was set at P < 0.05. Data are・・・・

**Revised, P19, line 323**

・・・・was set at p < 0.05. Data are・・・・

p.25, L.427, 429:

Fig 5D, F, p<0.01, Fig 5D, F, p<0.05 Isn't it "E" instead of "F"? please confirm.

**Answer:**

We thank the reviewer for this valuable comment. As pointed out, we have made a mistake. We apologize. This has been corrected as follows.

**Original, P25, line 427**

・・・the SAMR1 group (Fig 5D, F, p < 0.01).

**Revised, P25, line 433**

・・・the SAMR1 group (Fig 5D, E, p < 0.01).

**Original, P25, line 429**

・・・in IL-1RA expression (Fig 5D, F, p < 0.05).

**Revised, P25, line 435**

・・・in IL-1RA expression (Fig 5D, E, p < 0.05).

Materials and methods, Behavioral test:

Mice are nocturnal, so isn't it more reliable to perform behavioral tests at night?

**Answer:**

We thank the reviewer for this valuable comment. As per your comment, conducting tests during the daytime, while mice are nocturnal, may lead to incorrect conclusions at first glance. However, it is difficult to conduct tests at night in animal houses where animals are kept. Animals from other courses were also kept there, which may have affected the other studies. Under these restrictions, behavioral studies were conducted during the daytime. We have previously reported studies using SAMP8, and all behavioral studies were conducted during the day. Therefore, we considered that there were no problems with testing behavioral studies during the daytime in this study. We have added this information in the Methods section.

***Motor function test***

**Original, P11, line 176**

・・・was used for analysis. Tests were conducted between 10:00 a.m. and 1:00 p.m.

**Revised, P11, line 177**

・・・was used for analysis. Referring to previous studies [16–18], the tests were conducted between 10:00 am and 1:00 pm.

***Muscle strength grip-strength test***

**Original, P12, line 185**

・・・calculated. The test was conducted between 10:00 a.m. and 1:00 p.m.

**Revised, P12, line 186**

・・・calculated. Referring to previous studies [16–18], the test was conducted between 10:00 a.m. and 1:00 p.m.

***Behavioral test***

**Original, P12, line 197**

・・・was calculated. Tests were conducted between 10:00 a.m. and 5:00 p.m.

**Revised, P12, line 199**

・・・was calculated. Referring to previous studies [16–18], the tests were conducted between 10:00 a.m. and 5:00 p.m.

***Treadmill fatigue test***

**Original, P13, lines 206–207**

・・・was recorded. The test was conducted between 10:00 a.m. and 5:00 p.m.

**Revised, P13, line 210-212**

・・・was recorded. Referring to previous studies [16–18], the test was conducted between 10:00 a.m. and 5:00 p.m.

Materials and methods, Histology and immunohistochemistry:

There is no description of the location of the coronal section of the brain. It is usually written as how many mm forward from bregma.

**Answer:**

We thank the reviewer for this valuable comment. In this case, a coronal section was made 1 mm cephalad from the bregma. An addition has been made to the relevant section.

***Materials and Methods, Tissue preparation***

**Original, P14, line 222-223**

・・・embedded in paraffin. The paraffin-embedded brain tissue was prepared as 4 μm-thick coronal sections.

**Revised, P14, line 228-229**

・・・embedded in paraffin. The paraffin-embedded brain tissue was prepared as 4 μm-thick coronal sections. The coronal section was obtained 1 mm cranial from the bregma.

Figure 1:

The text compares SAMR and SAMP. However, the way graph "B" is drawn is not drawn as graph "D." Please check the graphs of other figures again. It is a horizontal bar that shows the p value.

**Answer:**

We thank the reviewer for this valuable comment. As you pointed out, the horizontal bars indicating the p-values in the graph are inconsistent. We apologize. The graph has been corrected as follows:

**Original Fig.1 C (left) & D (right)**	**Revised Fig.2 C (left) & D (right)**

Figure 4:

Regarding “A”, there was no difference between the three groups, is there any particular need to show it as a photograph? Also, why not show the positive cells with an arrow head if you want to show it?

**Answer:**

We thank the reviewer for this valuable comment. Neuroinflammation is associated with microglial activation and several NYT components are known to inhibit microglial activation. Therefore, the number of activated microglia was observed by immunohistochemical staining. Interestingly, in this study, despite the fact that microglial activation did not change, changes in cytokine expression were observed, and the images are displayed. Positive cells are indicated by arrowheads.

**A**

**R1C**

**P8C**

**P8N**

**Striatum**

**Cortex**
